# Importance of annexin V N-terminus for 2D crystal formation and quick purification protocol of recombinant annexin V

**DOI:** 10.1371/journal.pone.0278553

**Published:** 2022-12-22

**Authors:** Ryusei Yamada, Tran Ngoc Trang, Holger Flechsig, Toshiki Takeda, Noriyuki Kodera, Hiroki Konno

**Affiliations:** 1 College of Science and Engineering, School of Biological Science and Technology, Kanazawa University, Kanazawa, Japan; 2 Graduate School of Frontier Science Initiative, Kanazawa University, Kanazawa, Japan; 3 WPI Nano Life Science Institute (WPI-NanoLSI), Kanazawa University, Kanazawa, Japan; 4 College of Science and Engineering, School of Natural System, Kanazawa University, Kanazawa, Japan; George Washington University, UNITED STATES

## Abstract

Annexin V forms trimeric structures which further assemble into two-dimensional crystal (2D crystal) lattices on negatively charged phospholipid bilayer in a Ca^2+^-dependent manner. It is also known that annexin V 2D crystals show two types of symmetric patterns with six-fold symmetry (*p*6) and three-fold symmetry (*p*3). The *p*6 lattice also contains additional trimers in the gaps between the *p*6 axes, which are also referred to as non-*p*6 trimers because they do not participate in the formation of the *p*6 lattice. We here show that the annexin V N-terminal has significant influence on 2D crystal formation using high-speed atomic force microscopy (HS-AFM) observations. We also present a quick purification method to purify recombinant annexin V without any residual affinity tag after protein purification in ~3h.

## Introduction

**Fig 1:**
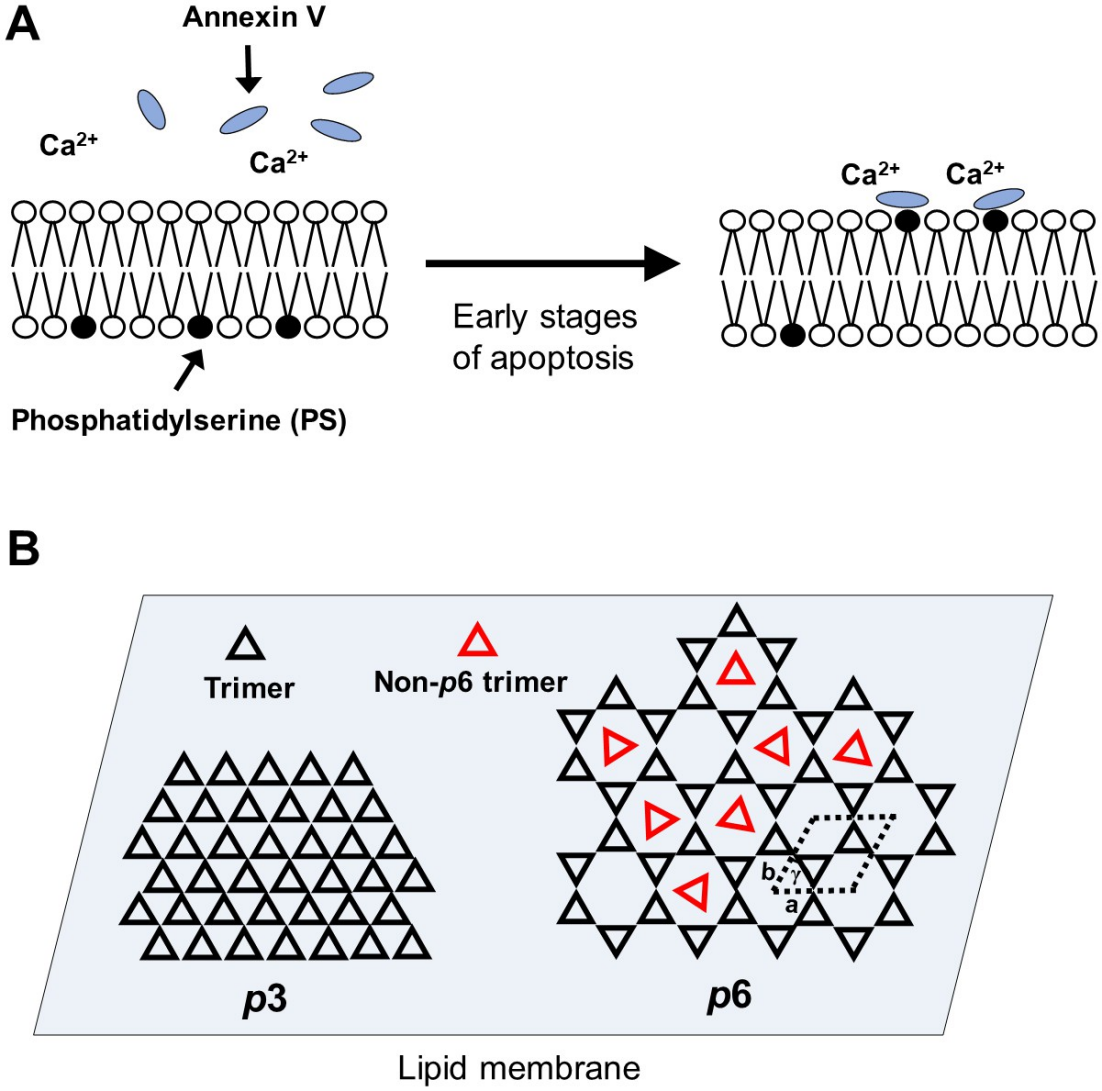
Schematic model of annexin V binding on cell membrane and two types of annexin V 2D-crystal. (A) When the phosphatidylserines (PS) flip to the extracellular surface of the cell, annexin V binds to PS in Ca^2+^-dependent manner. (B) Annexin V 2D crystals show two types of symmetric patterns with three-fold symmetry (*p*3) and six-fold symmetry (*p*6). The *p*6 lattice also contains additional trimers (red triangle) in the gaps between the *p*6 axes, which are also referred to as non-*p*6 trimers because they do not participate in the formation of the *p*6 lattice. The 2D crystals of annexin V assembled into a lattice with *p*6 symmetry (unit cell: a = b = 17.7 nm, γ = 60°).

Annexin V exhibits tight binding to cover the negatively charged phospholipids such as phosphatidylserine (PS) on the cell membrane [1, 2]. It is known that annexin V is involved in local regulation of coagulation when PS in the inner layer of the cell membrane is exposed to the outer layer of the cell membrane (flip-flop) by apoptosis, activation of monocytes, platelets, and vascular endothelial cells [3, 4] (Fig 1A). Since binding of Annexin V to the cellular membrane upon PS exposure occurs earlier than the DNA fragmentation, it can be used to detect cells in the early stages of apoptosis [1]. To detect early phase of apoptosis in real time *in vivo*, fluorescence such as FITC conjugated annexin V is widely used (Fig 1A) [5]. However, conjugation requires large amounts of protein in general. So far, there are a numerous reports showing efficient purification methods of recombinant annexin V [6–10]. Recombinant annexin V is purified by tag-dependent or tag-free purification methods. In the major case of tag-free purification, a combination of several different chromatographic techniques such as ion-exchange, hydrophobic interactions and size exclusion chromatography is required. Tag-dependent purification can be achieved by a single-step procedure, but the tag must be removed if necessary. The tag is removed from the purified protein by introducing a protease cleavage sequence between the tag and the target protein. Therefore, it is necessary to separate the protease from the protein after removing the tag, resulting in a more time-consuming purification with more steps. We here propose a high-throughput purification protocol of annexin V without any residual tag after protein purification in ~3h.

It is known that Annexin V forms trimeric structures which further assemble into two-dimensional crystal (2D crystal) lattices on negatively charged phospholipid bilayer in a Ca^2+^-dependent manner [11–13]. Since annexin V 2D forms a relatively large lattice, quick purification of annexin V would be very useful not only for elucidating the formation mechanism of its 2D crystal, but also for calibrating the AFM scanner in the XY direction. The annexin V 2D crystals show two types of symmetric patterns with six-fold symmetry (*p*6) and three-fold symmetry (*p*3) [11–13] (Fig 1B). The *p*6 lattice also contains additional trimers in the gaps between the *p*6 axes, which are also referred to as non-*p*6 trimers because they do not participate in the formation of the *p*6 lattice. In this study, we also demonstrate the importance of the annexin V N-terminus for 2D crystal formation.

## Results

### HS-AFM observation of annexin V 2D crystal using N-terminus His-tagged recombinant annexin V

**Fig 2:**
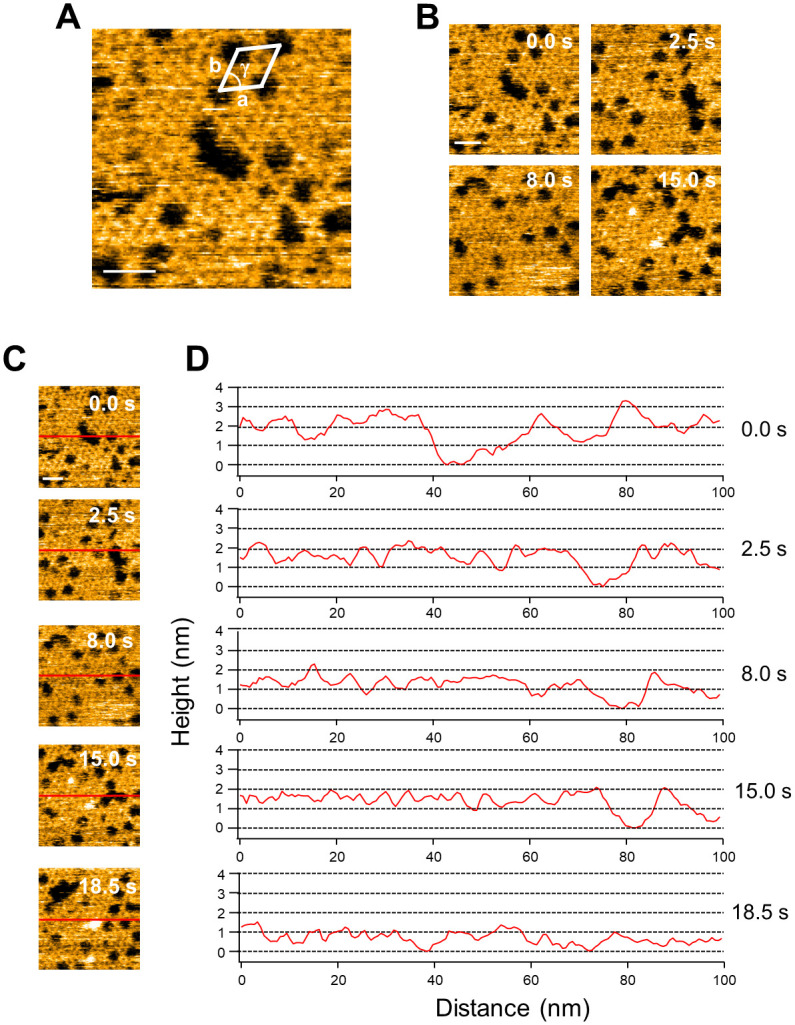
Effect of N-terminus His-tag on the annexin V 2D crystal formation. (A) HS-AFM imaging of 2D crystal of N-terminus His-tagged Annexin V. The operational parameters were scan range, 100 × 100 nm (150 × 150 pixels); scan rate, 0.5 s/frame; and z-scale, 5 nm, bar 20 nm. The 2D crystals of the annexin V assembled into a lattice with *p*6 symmetry (unit cell: a = b = 17.7 nm, γ = 60°). (B) HS-AFM image sequence of N-terminal His-tagged Annexin V 2D crystal. (C) Selected snapshots in which heights of the 2D crystal were measured (red lines). (D) Corresponding line profiles of 2D crystal heights at the indicated times.

**Fig 3:**
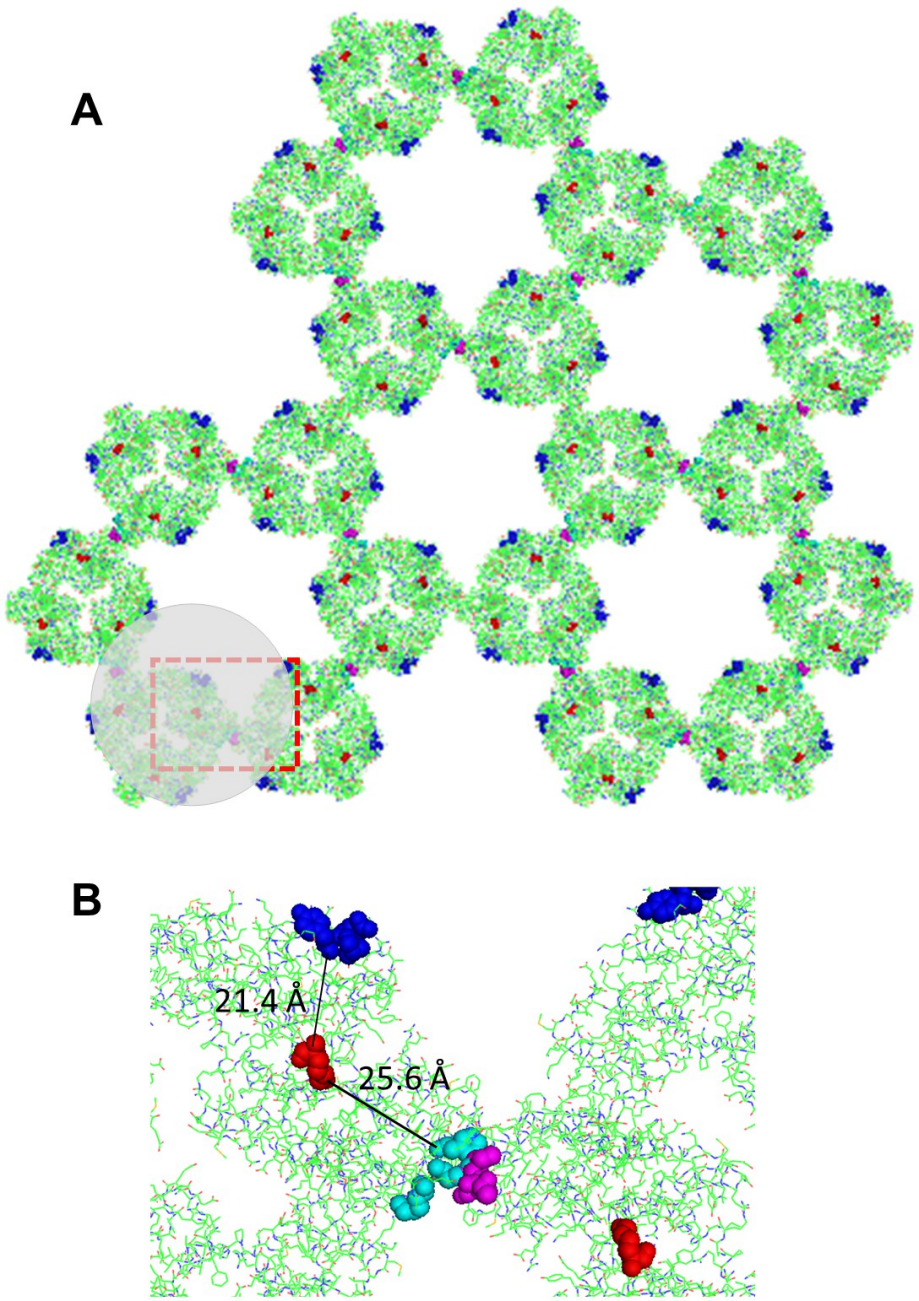
Molecular model of the annexin V p6 lattice (see methods). (A) Top view of the 2D crystal model in the membrane-bound perspective. In each trimer, the N-termini of monomers are indicated by red spheres. S295, Y297 and Q298 (corresponding to S295, Y297 and S298 in human annexin V) of the fourth annexin repeat are shown as blue spheres. T215, I216, S217 of the third repeat in annexin V are shown as magenta spheres. K174, Q177, F180 (corresponding to Q174, Q177, F180 in human annexin V) of the third repeat in the adjacent annexin V are shown in cyan spheres. The range where a 19 amino acids long peptide (0.35 nm × 19 = 6.65 nm) from N-terminus would affect 2D crystal formation is shown in gray. (B) Magnified image of the area marked by the red rectangle in (A).

We first expressed annexin V with a N-terminus His-tag in *E*. *coli* cells and purified the protein. The residual amino acids at the N-terminus of annexin V were 19 amino acids including 6×His-tag and enterokinase cleavage sites (S1 Fig). For AFM observation, supported lipid bilayers containing 20% phosphatidylserine were formed on the mica substrate in a buffer containing 2 mM Ca^2+^. After that, N-terminus His-tagged annexin V was added. The 2D annexin V crystals assembled in a lattice of *p*6 symmetry (unit cell: a = b = 17.7 nm, γ = 60°) with the trimer as the smallest building unit (Fig 2A). 2D crystals of *p*6 are also known to contain additional trimers in the gaps between the *p*6 axes, which are also referred to as non-*p*6 trimers because they do not participate in the formation of the *p*6 lattice. As described above, AFM observations confirmed that annexin V with an N-terminus His-tag forms *p*6 crystals, but, as we find, non-*p*6 trimers were frequently lacking. (Fig 2A). Moreover, non-*p*6 trimers were also observed to frequently change positions within the lattice (Fig 2B). The time course of height line profiles at the same position in the 2D crystal varied widely over a short time interval (Fig 2C and 2D). These results indicate that the N-terminus His-tag of annexin V has a significant effect on interactions between *p*6 trimers and the non-*p*6 trimer. We also performed size exclusion chromatography to evaluate the stability of His-tagged Annexin V (S2 Fig). The His-tagged Annexin V eluted as major single peaks with retention time almost corresponding to the monomer. When the Annexin V was unstable and totally or partially denatured after Ni-NTA purification, aggregation of Annexin V would prevent monodisperse, and the elution profile would not have shown single peak in size exclusion chromatography. Therefore, we conclude that His-tag does not affect to stability of Annexin V monomer but His-tag affect to the interaction between Annexin V molecules in 2D lattice. To discuss the influence of the N-terminus structure on the stability of the 2D lattice, we constructed a molecular model based on the Annexin V monomer (PDB ID, 1ALA) (Fig 3A). It is reported that the non-*p*6 trimer interacts with S295, Y297 and Q298, corresponding to S295, Y297 and S298 in human annexin V, of the fourth annexin repeat in the adjacent *p*6 trimer [14] (Figs 2A and 3B). Indeed, the N-terminus was present near S295, Y297 and Q298 (Fig 3A and 3B). AFM observations also revealed that the formation of the *p*6 lattice by N-terminus His-tagged annexin V was incomplete, although this was found less striking as compared to the lack of non-*p*6 trimers from the 2D crystal (Fig 2A–2C and S1 Movie). For the formation of the *p*6 lattice, it is known that interactions between T215, I216, S217 of the third repeat in annexin V and K174, Q177, F180 of the third repeat in an adjacent annexin V are important [14] (Fig 3B). In this study 19 amino acids including 6×His were introduced at the N-terminus. Assuming an average length of 0.35 nm for one amino acid, the total length of the 19 amino acids is 6.65 nm. Since the distance from the N-terminus to the interaction site between annexins, which is important for 2D lattice formation, is 2~2.5 nm (Fig 3B), the length of 19 amino acids is sufficient to affect the interactions between annexins in the 2D lattice. Therefore, with very short tags (~2 nm) at the N-terminus side, it may be possible to observe stable 2D crystals even with the tags still attached. Taken together, residual amino acids at the N-terminus of annexin V were found to destabilize the 2D crystal by affecting both the insertion of non-*p*6 trimers into the 2D crystal and interactions between *p*6 trimers.

### Purification of annexin V without any residual affinity tag at the N-terminus

**Fig 4:**
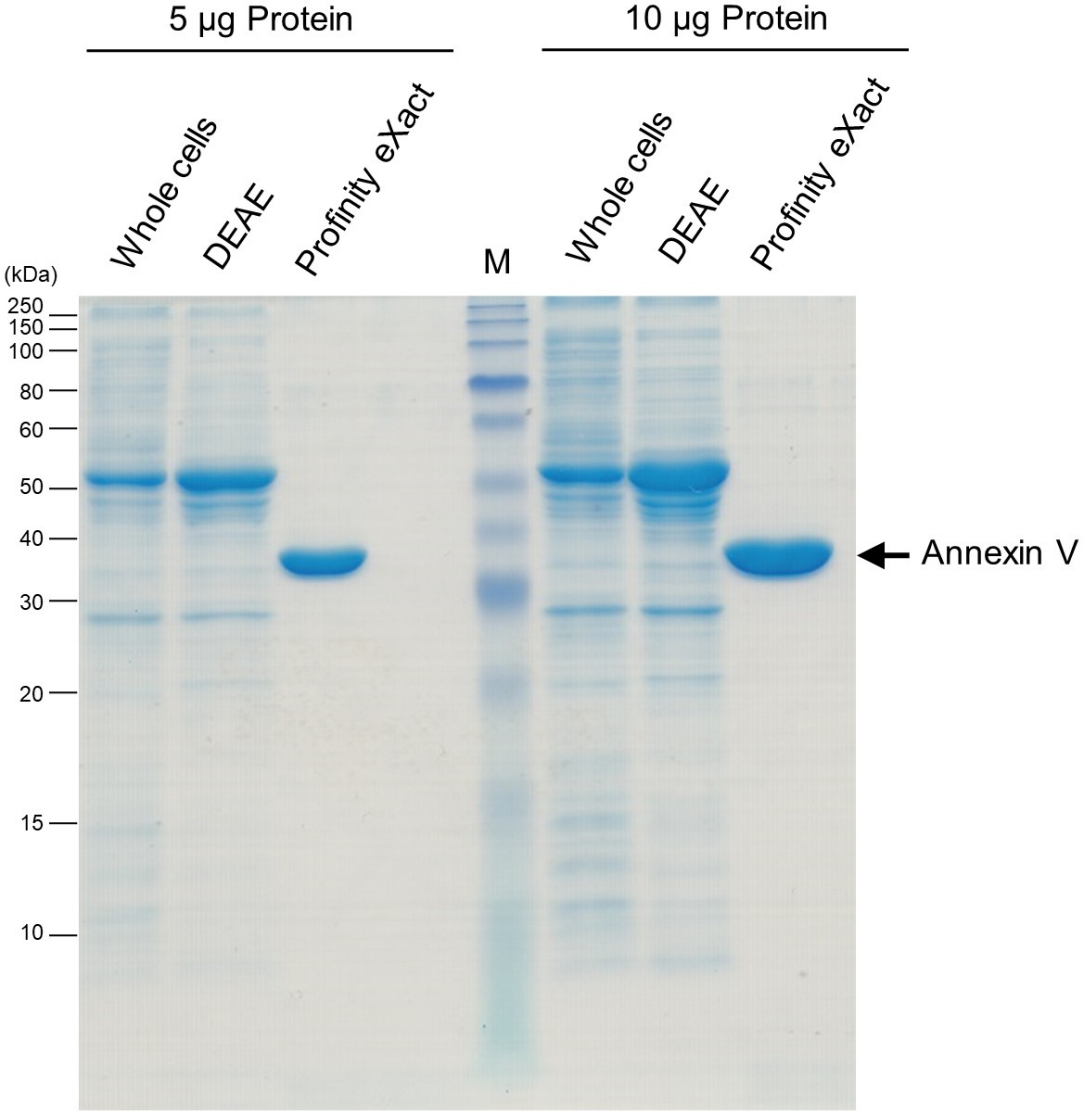
Quick preparation of non-tagged annexin V. The purity of recombinant annexin V was analyzed by 15% (w/v) SDS-PAGE. M, molecular weight maker; Whole cells, whole proteins of the cell lysate; DEAE, annexin V protein after DEAE chromatography; Profinity eXact, annexin V protein after Profinity eXact purification. A band of ≈50 kDa was observed as a main band after DEAE chromatography, but after the tag was removed during elution procedure, a band consistent with the molecular weight of annexin V (36 kDa) was detected. The band position of annexin V after Profinity eXact purification was indicated by the arrow.

As described above, 2D crystals formed by N-terminus His-tagged annexin V was structurally very unstable. Therefore, we tried to prepare annexin V without any residual affinity tag. Although, affinity tag removal after protein purification is widely used, in most cases the tag is removed from the purified protein by introducing a protease cleavage sequence between the tag and the target protein. However, in the case of trypsin, an extra sequence of three amino acids remains on the target protein side even after cleavage. There are several proteases which does not have any extra sequence such as Factor X, but even in that case, it is necessary to separate the protease from the protein after removing the tag, resulting in a more time-consuming purification with more steps. Therefore, we attempted to purify annexin V suitable for 2D crystal formation using an *E*. *coli*-based expression and purification system (Profinity eXact fusion-tag system) [15, 16]. In this system, a stability improved subtilisin protease immobilized on the column strongly binds to an affinity tag which is fused at the N-terminus of the target protein [15, 16]. The protease then undergoes specific cleavage controlled by triggering anions such as F^−^ in the buffer [15, 16]. Then, the tag is removed from the target protein directly on the column, resulting in a highly purified target protein with the native N-terminus within a single step. Before the removal of the profinity tag a band of ≈50 kDa was observed as a main band, but after the tag was removed during elution procedure, a band was detected at a position consistent with the molecular weight of annexin V (36 kDa) (Fig 4). By using the above purification methods, we could obtain 1.5 mg of annexin V per 1 g of *E*. *coli* cells in ~3 h. The purification step with DEAE column could be omitted. However, to prolong the life of Profinity eXact^™^ resin, we recommend performing the DEAE purification step.

### HS-AFM observation of the 2D crystal using annexin V without any N-terminus residual amino acids

**Fig 5:**
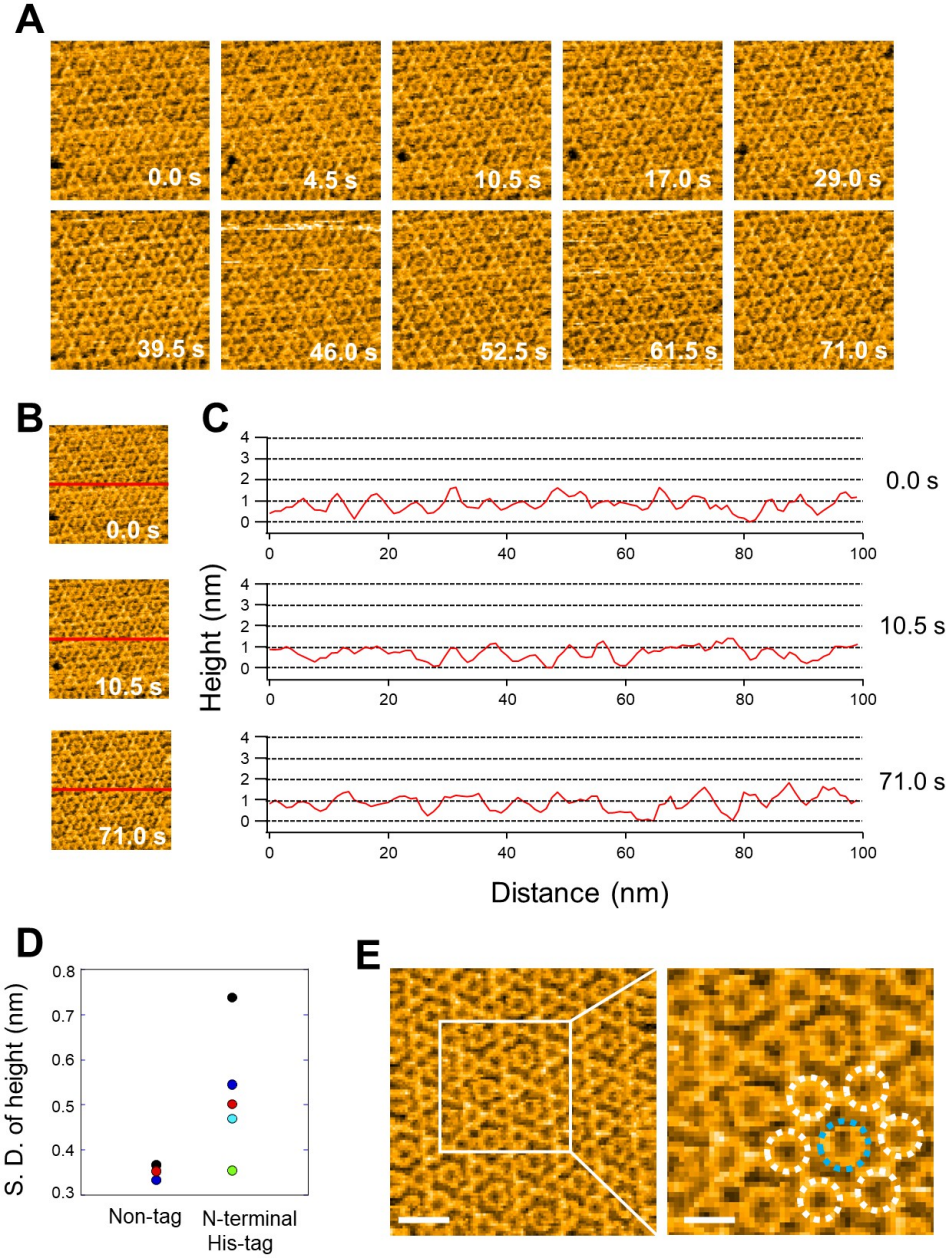
2D crystal formation by non-tagged annexin V. (A) HS-AFM image sequence of the 2D crystal of annexin V without any residual amino acids. The operational parameters were scan range, 100 × 100 nm (106 × 106 pixels); scan rate, 0.5 s/frame; and z-scale, 5 nm, bar 20 nm. (B) The same image sequence of 2D crystal at 0 s, 10.5 s and 71.0 s as in (A) indicating where heights of the 2D crystal were measured (red lines). (C) Corresponding line profiles of 2D crystal heights at the indicated times. (D) Standard deviation (S. D.) of annexin 2D crystal heights of N-terminus His-tagged and non-tagged recombinant annexin V. S. D. values of non-tagged recombinant annexin V were calculated from the height line profiles in (C). S. D. values of N-terminus His-tagged annexin V were calculated from the height line profiles in (Fig 2D). (E) Left panel, another example of HS-AFM imaging of 2D crystal of annexin V without any residual amino acids. The operational parameters of HS-AFM observation were the same as in (A). Right panel, magnified image of the region indicated by white lines in the left panel, where trimers forming the *p*6 lattice are marked by white circles; the non-*p*6 trimer in the 2D crystal is marked by a blue circle.

**Fig 6:**
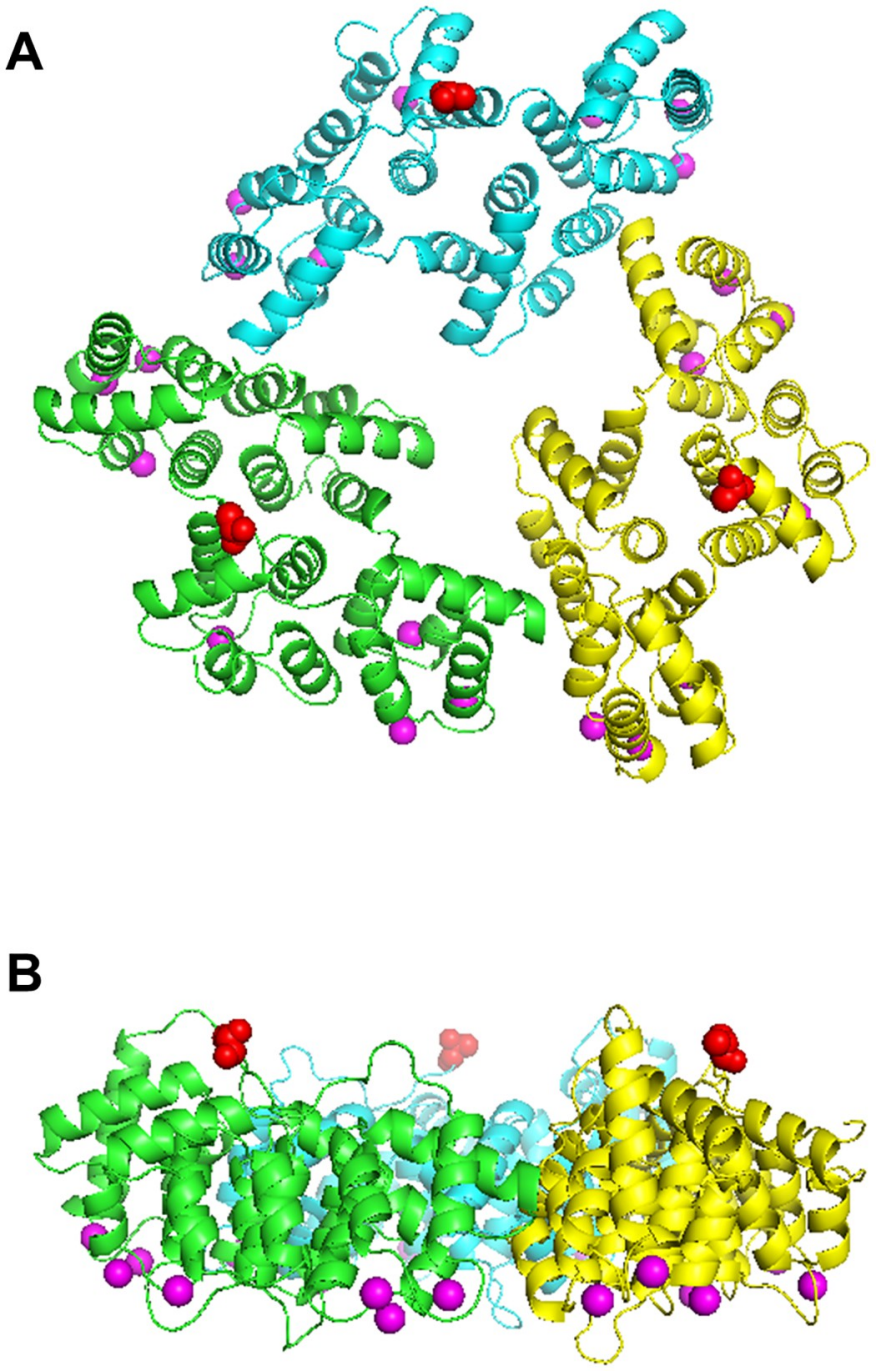
Structure of annexin V trimer (PDB:2IE7). (A) Top view of annexin V trimer from N-terminus side. Monomers in the annexin V trimer are colored in green, cyan, and yellow, respectively. The N-terminus of each monomer is indicated by red spheres. Ca^2+^ ions in the trimer are shown in purple. (B) Side view of the annexin V trimer in (A). The N-termini (red spheres) are located on the opposite side of Ca^2+^ (purple).

When we have grown 2D crystals by annexin V without any residual amino acids, the formation efficiency of 2D crystal was significantly increased compared to that of N-terminus His-tagged annexin V (Fig 5A and S2 Movie). There was almost no deletion of the non-*p*6 trimer over a long period of time (Fig 5A–5C). Indeed, standard errors of the 2D crystal heights of non-His-tagged annexin V over the observation period were very stable (Fig 5D). On the other hand, the variation of standard errors within the observation time was very large for N-terminus His-tagged annexin V (Fig 5D). In addition, AFM images with high resolution were obtained probably due to the high stability of the 2D crystals (Fig 5E). These results indicate that the N-terminus sequence of annexin V is critical for the formation and stability of 2D crystals, especially regarding the insertion of non-*p*6 trimers into the *p*6 lattice. It is also known that the presence of non-*p*6 trimers is sensitive to changes in Ca^2+^ concentration, and their dissociation from the 2D lattice occurs when the Ca^2+^ concentration is gradually reduced from the solution [14]. However, the N-terminus of annexin V is located at the opposite side of Ca^2+^ and lipid binding sites (Fig 6A and 6B). Therefore, the instability of the 2D crystal by N-terminus His-tagged annexin V observed in our study seems unrelated to reduced affinity with Ca^2+^ or lipids. As an alternative explanation we found that slight differences in the N-terminus conformation can significantly alter the assembly state of annexin V 2D crystals.

## Discussion

In this study, we showed that the annexin V N-terminal has significant influence on 2D crystal formation using HS-AFM. We also presented a quick purification method to purify recombinant annexin V without any residual affinity tag after protein purification. Based on the results, it would be better to use annexin V which does not contain any tag sequences such as His-tag or FLAG-tag at the N-terminus to prevent an experimental artifact not only in AFM observations but also other biological experiments. Indeed, untagged annexin V was often used to investigate membrane-association and self-assembly processes of annexin V [14, 17, 18], though the C-terminus might not so much affect assembly since annexin V containing His-tag on its C-terminus forms 2D crystals even with the His-tag [12]. Our results also indicate the important role of N-terminus regions in annexins to maintain functional diversity in the cell by altering the assembly state. For example, Ca^2+^-dependent membrane binding detaches the N-terminus of annexin I from the annexin third annexin repeat [19]. The detached N-terminus can interact with other proteins, especially the S-100 protein family. In addition, the N-terminus is often subject to post-translational modification that allow for further signal transduction [19]. Therefore, the N-terminus of the annexin group has a key role of their functional diversity. In the future, it may be possible to artificially modify the functions of annexins by altering the N-terminus sequence.

## Materials & methods

### Expression and purification of His-annexin V

DNA fragments coding 320 amino acids of Homo Sapiens annexin V and 19 amino acids containing His-tag and enterokinase cleavage sequences at N-terminus on annexin V were synthesized as a codon optimized artificial gene (GenScript, NJ, US) (S1 Fig). The products were subsequently ligated into the pET23a vector with NdeI and BamHI restriction enzymes. The BL21 (DE3) *E*. *coli* ells harboring annexin V expression plasmids were cultured in LB medium containing 40 μg/ml kanamycin up to an OD_600_ of 0.5–0.7 at 37°C, and protein expression was then induced by the addition of 0.1 mM IPTG at 19°C for 15–18 h. Cells were collected by centrifugation, suspended in 50 mM NaPi (pH 8.0), 300 mM NaCl, and 20 mM Imidazole, and disrupted using a French press. The supernatant obtained after lysate centrifugation was subsequently applied to the Ni-NTA superflow column (QIAGEN, Hilden, DE). The column was washed with 10 column volume of the same buffer. The annexin V was then eluted with 50 mM NaPi (pH 8.0), 300 mM NaCl, 200 mM imidazole. The elution fraction was concentrated using Amicon^®^ Ultra Centrifugal Filters 30,000 MWCO (Millipore, MA, US). The concentrated sample was applied to the Superdex 200 gel filtration chromatography column equilibrated with 20 mM Tris-HCl (pH 7.5), 150 mM NaCl with a 0.5 ml/min flow rate, and elution was monitored at 280 nm. The concentration of the annexin V was quantitated with the Bradford method (Bio-rad, CA, US), and the product was stored at −80°C until use.

### HS-AFM observations

Lipids (dioleoylpho-sphatidyl-choline (DOPC), dioleoylphosphatidyl-serine (DOPS), 1,2-dioleoyl-sn-glycero-3-phosphoethanolamine-N-(biotinyl) (Biotinyl-DOPE) were purchased from Avanti polar lipids. Three μl of liposome made with the lipid composition of DOPC:DOPS:biotinyl-DOPE = 7:2:1 were put on mica and incubated for 10 min, then washed 10 mM HEPES-NaOH pH 7.0, 2 mM CaCl_2_, 150 mM NaCl. Then, 1 μM annexin V was added on the lipids-coated mica and incubate for 10 min. AFM imaging of annexin V 2D crystal was performed in solution using a laboratory-built HS-AFM setup [20–22]. Imaging was carried out in the tapping mode, using small cantilevers (BL-AC10DS-A2 or custom-made BL-AC7DS-KU4, Olympus, Tokyo, Japan). The cantilever free oscillation amplitude was ~1.5 nm, and the set-point amplitude was 80–90% of the free oscillation amplitude. The Imaging rate, scan size, and feedback parameters were optimized to enable visualization with a minimum tip force. The obtained data were analyzed by custom software “Falcon viewer” prepared by Prof. T. Uchihashi (Nagoya university).

### Expression, and purification of annexin V by Profinity eXact^™^ system

To ligated annexin V gene into the pPAL7 expression vector (Bio-rad, CA, US), the artificial synthesized gene of Homo Sapiens annexin V as described above was amplified by PCR with the following primers: 5′-GGCGGGGCTCTTCAAAGCTTTGATGGCGC AAGTTCTGC-3′ (SapI), 5′-CGCGGCGGATCCTTAGTCATCTTCACCGC-3′ (BamHI). Then, the PCR products ligated into the pPAL7 vector with SapI and BamHI restriction enzymes. The BL21 (DE3) *E*. *coli* cells harboring annexin V expression plasmids were cultured in LB medium containing 100 μg/ml Ampicillin up to an OD_600_ of 0.5–0.7 at 37°C, and protein expression was then induced by the addition of 0.1 mM IPTG at 19°C for 15–18 h, and the cells were collected by centrifugation. *E*. *Coli* cells expressing annexin V were suspended in 50 mM NaPi (pH 7.2), 1 mM EDTA, and disrupted using a French press. The supernatant obtained after lysate centrifugation was subsequently applied to a Toyopearl DEAE-650M (Tosoh, Tokyo, JP) column equilibrated with the same buffer. The column was washed with 10 column volume of the same buffer, and the flow through fraction containing annexin V was applied to a Profinity eXact^™^ (exact affinity cleavage technology, Bio-rad, CA, US) protein purification column equilibrated with 100 mM NaPi (pH 7.2) in cold room (4°C). The column was washed with 15 column volume of the same buffer in cold room. Next, apply 5 column volume of elution buffer, 100 mM NaPi (pH 7.2) and 100 mM NaF, to plugged column, and mix the resin with the elution buffer by using a pipette and incubated the column in the elution buffer for 30 min at 25°C. To elute the annexin V from the column, remove the column’s plug and allow elute containing the annexin V to gravity drain into collection tube. The eluted fractions were concentrated and exchanged the buffer to 40 mM Tris-HCl (pH 7.5), 150 mM NaCl, 1 mM DTT, 0.05% NaN_3_, and stored at −80°C until use.

### Modeling of annexin V *p*6 2D crystal

Construction of the annexin V 2D-lattice molecular structure was based on our previous work [18], where simulation atomic force microscopy (BioAFMviewer software, Ref. [23]) was employed to reconstruct the atomistic structure of the hexameric arrangement of annexin V trimers from a correlation-averaged experimental AFM image of the *p*6 lattice (see Ref. [18] for details). The molecular model of the 2D crystal shown in Fig 3 was assembled by applying translations of the hexamer structure using the parameters of the *p*6 lattice geometry.

## Supporting information

S1 Fig Amino acid sequence of N-terminus His-tagged annexin V. The sequence of 6×His and enterokinase cleavage site is shown in red and blue, respectively. Amino acids except His-tag and the cleavage site in the residual sequence are shown in gray.
(TIF)

S2 Fig Elution profile of size exclusion chromatography of N-terminus His-tagged annexin V. N-terminus His-tagged Annexin V after Ni-NTA purification was applied size exclusion chromatography as described in materials and methods. The arrows indicate elution times of molecular weight maker proteins, Thyroglobulin for 669 kDa, Ferritin for 440 kDa, Aldolase for 158 kDa, Conalbumin for 75 kDa and Ovalbumin for 43 kDa, respectively.
(TIF)

S1 Movie HS-AFM movie showing 2D lattice of N-terminus His-tagged annexin V. Scan size, 100×100 nm. Imaging rate, 2 fps. The movie is played at ×4 higher speed. Total recording time, 40 sec.
(MP4)

S2 Movie HS-AFM movie showing 2D lattice of non-tagged annexin V. Scan size, 100×100 nm. Imaging rate, 2 fps. The movie is played at ×4 higher speed. Total recording time, 71 sec.
(MP4)

S1 Data Plot data for size exclusion chromatograpu of His-tagged annexin V. (XLSX)

S2 Data Plot data for the standard deviation (S.D.) in Fig 5D. (XLSX)

S3 Data Original file of SDS-PAGE gel in Fig 4. (JPG)
