# Supplementary figures and images for: Importance of annexin V N-terminus for 2D crystal formation and quick purification protocol of recombinant annexin V
Source: PLoS One. 2022 Dec 22;17(12):e0278553. doi: 10.1371/journal.pone.0278553 (PMC9778525; doi:10.1371/journal.pone.0278553)

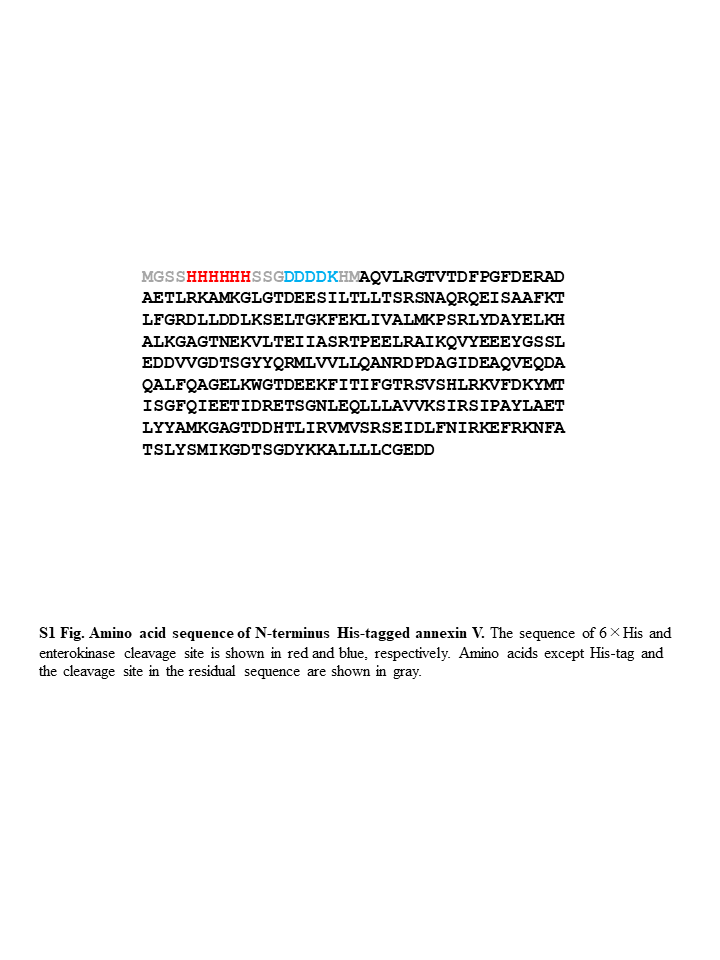

Supplement: S1 Fig — The sequence of 6×His and enterokinase cleavage site is shown in red and blue, respectively. Amino acids except His-tag and the cleavage site in the residual sequence are shown in gray. (TIF) [file pone.0278553.s001.TIF]

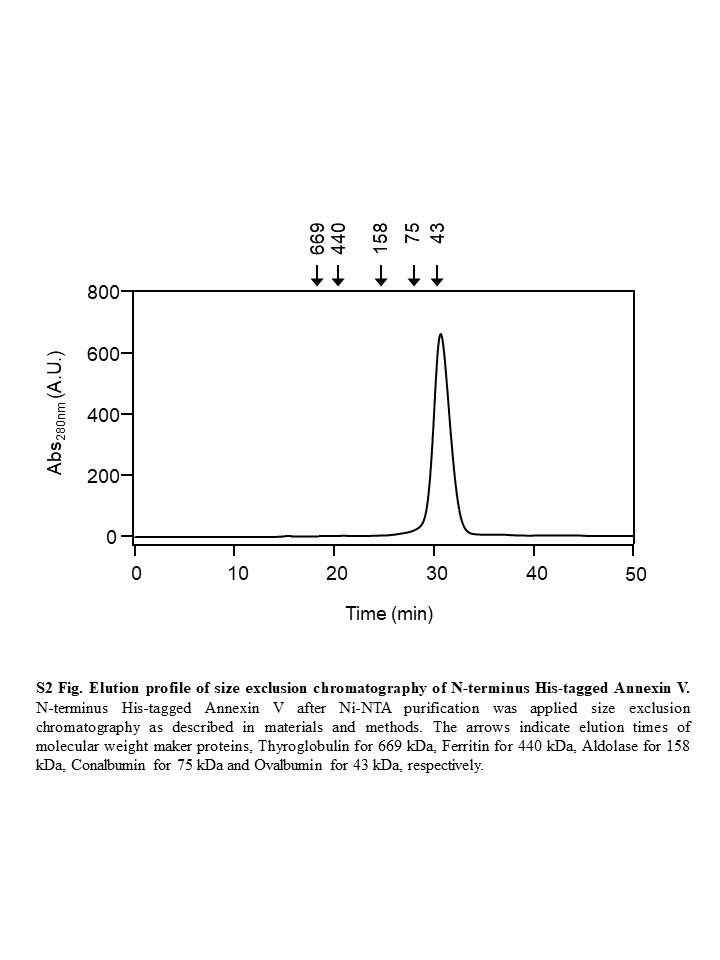

Supplement: S2 Fig — N-terminus His-tagged Annexin V after Ni-NTA purification was applied size exclusion chromatography as described in materials and methods. The arrows indicate elution times of molecular weight maker proteins, Thyroglobulin for 669 kDa, Ferritin for 440 kDa, Aldolase for 158 kDa, Conalbumin for 75 kDa and Ovalbumin for 43 kDa, respectively. (TIF) [file pone.0278553.s002.TIF]

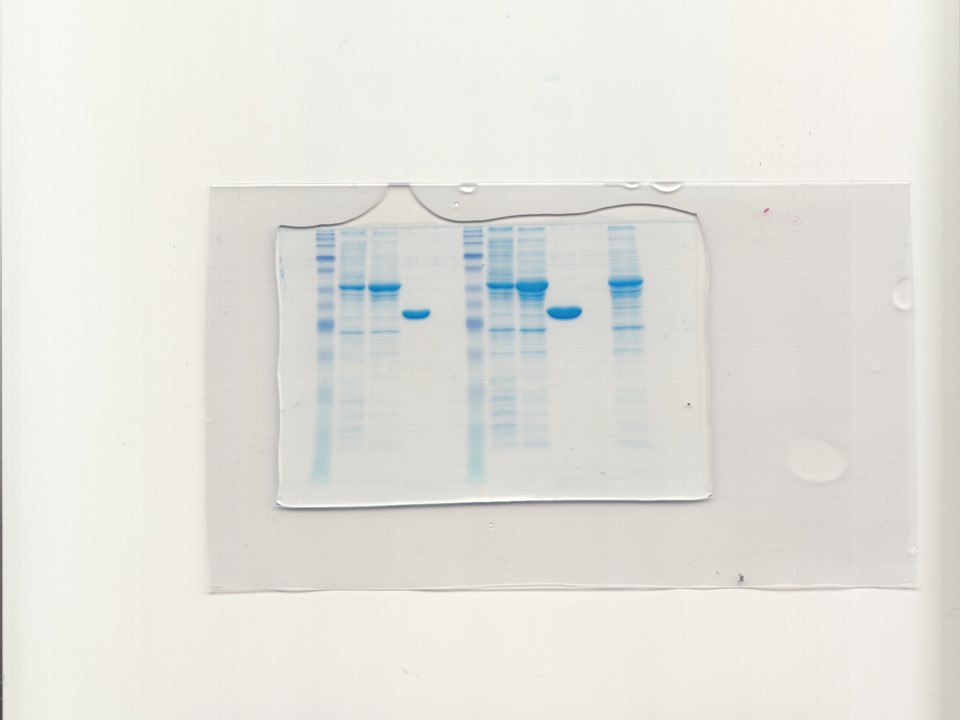

Supplement: S3 Data — (JPG) [file pone.0278553.s007.jpg]
